# Deriving emission factors for mangrove blue carbon ecosystem in Indonesia

**DOI:** 10.1186/s13021-023-00233-1

**Published:** 2023-07-13

**Authors:** Daniel Murdiyarso, Haruni Krisnawati, Wahyu C. Adinugroho, Sigit D. Sasmito

**Affiliations:** 1 Center for International Forestry Research – World Agroforestry, Jl. CIFOR, Situgede, Bogor, 16115 Indonesia; 2 Department of Geophysics and Meteorology, IPB University, Bogor, 16680 Indonesia; 3 Research Center for Ecology and Ethnobiology, National Research and Innovation Agency, Bogor, Indonesia; 4 NUS Environmental Research Institute, National University of Singapore, 21 Lower Kent Ridge Road, Singapore, 119077 Singapore

**Keywords:** Aquaculture, Carbon stocks, Forest reference emission level (FREL), GHG fluxes, National Greenhouse Gas Inventories (NGGI), Mangrove restoration, Nature based climate solutions

## Abstract

**Background:**

Using ‘higher-tier’ emission factors in National Greenhouse Gas Inventories is essential to improve quality and accuracy when reporting carbon emissions and removals. Here we systematically reviewed 736 data across 249 sites (published 2003–2020) to derive emission factors associated with land-use change in Indonesian mangroves blue carbon ecosystems.

**Results:**

Four management regimes—aquaculture, degraded mangrove, regenerated mangrove and undisturbed mangrove—gave mean total ecosystem carbon stocks of 579, 717, 890, and 1061 Mg C ha^−1^ respectively. The largest biomass carbon stocks were found in undisturbed mangrove; followed by regenerated mangrove, degraded mangrove, and aquaculture. Top 100-cm soil carbon stocks were similar across regimes, ranging between 216 and 296 Mg C ha^−1^. Carbon stocks between 0 and 300 cm varied significantly; the highest values were found in undisturbed mangrove (916 Mg C ha^−1^), followed by regenerated mangrove (803 Mg C ha^−1^), degraded mangrove 666 Mg C ha^−1^), and aquaculture (562 Mg C ha^−1^).

**Conclusions:**

Using deep layer (e.g., 300 cm) soil carbon stocks would compensate for the underestimation of surface soil carbon removed from areas where aquaculture is widely practised. From a project perspective, deep layer data could secure permanence or buffer potential leakages. From a national GHG accounting perspective, it also provides a safeguard in the MRV system.

**Supplementary Information:**

The online version contains supplementary material available at 10.1186/s13021-023-00233-1.

## Background

Emission factors (EFs) are representative values or coefficients used when calculating anthropogenic emissions by sources, and removals by sinks of greenhouse gases (GHGs). The Intergovernmental Panel on Climate Change (IPCC) emission factor database contains default data, known as Tier 1, and data from peer-reviewed journals and other publications with higher tiers (i.e., Tier 2 and 3). Country-specific data are usually considered Tier 2, and those values obtained using the most detailed methods, like modelling, are considered Tier 3. The IPCC emission factor database is managed by the IPCC’s Task Force on National Greenhouse Gas Inventories, and supported by the National Greenhouse Gas Inventories Programme (https://www.ipcc-nggip.iges.or.jp/EFDB/main.php). The emission factor database becomes the main reference for countries where Tier 2 and 3 EFs are unavailable.

With National Greenhouse Gas Inventories (NGGI), best practice must abide by certain principles (e.g., transparency, accuracy, completeness, comparability, and consistency), specifically in the use of higher-tier activity data and EFs for land-based emissions monitoring. To reduce uncertainties in GHG emission reduction targets, countries or project developers may prefer to use higher-tier EFs, as they tend to offer higher levels of accuracy. Therefore, deriving country-specific or even site-specific higher-tier EFs can further improve the quality of the NGGI, and thus the credibility of national measuring, reporting and verification (MRV) processes [1].

Since the 2013 Supplement to the 2006 IPCC Guidelines for NGGI for Wetlands was published [2], just a few countries have adopted the guidelines for their national reporting. The Supplement, which was designed to address high-carbon reservoirs in wetlands, includes peatlands and mangroves. It follows the Agriculture, Forestry and Other Land Use (AFOLU) sector key categories analysis, under the 2006 IPCC Guidelines and its predecessor the 1996 IPCC Guidelines. The total ecosystem carbon stocks (TECS) is the sum of the following carbon pools: aboveground biomass carbon (AGBC), belowground biomass carbon (BGBC), dead organic matter (DOM), and soil organic carbon (SOC). To estimate anthropogenic emissions by sources and removals by sinks, the 2006 IPCC guidelines provides two approach options; the stock-difference approach and the gain–loss approach [3]. Both produce comparable estimates. Although countries such as Australia are interested in including blue carbon in their national emissions reduction policy, the use of the guidelines around this is still unclear [4]. Indonesia, meanwhile, has been incorporating mangrove into its improved Forest Reference Emission Level (FREL), which was initially submitted to the Secretariat of the United Nations Framework Convention on Climate Change (UNFCCC) in 2016.

Indonesia is home to around 3 million hectares of mangroves—almost a quarter of world’s mangrove area is found in this archipelagic country with its 900,000 km coastline [5]. While Indonesian mangrove is facing tremendous pressure from the development of aquaculture and agriculture [6], this carbon-rich coastal forest is also one of the key ecosystems for nature-based climate solutions [7]. Mangroves in Indonesia, which are dominated by *Rhizophora* spp and are predominantly located in estuarine and deltaic coastal settings, store considerably high TECS, with means of 1083 ± 378 Mg C ha^−1^ [6]. Unlike most terrestrial forests, mangroves are halophyte coastal vegetation, tolerating high salinity and other harsh conditions in the root environment, making these coastal forests strong contenders in a world with rising sea levels [8]. This is probably the reason mangroves have a high turnover of fine root production [9]. As a result, together with the two other blue carbon ecosystems—seagrasses and saltmarshes [10]—mangrove is one of the ecosystems with the highest carbon burial rates; as much as 20 times more than terrestrial forests [11], contributing to the high proportions of carbon stocks in their soil carbon pools [6, 12].

The source of mangrove soil carbon, especially in the first meter or top soil, is primarily driven by the tidal transport of allocthonous sediment; while in-situ or autochtonous sequestration is predominantly recalcitrant carbon [13]. The use of the top 1 m estimate, however, may cause a 40% underestimation of TECS [14]. The fact that soil carbon dominates TECS by up to 80–90% [6, 15] suggests that the soil excavation that is generally practiced in the development of aquaculture should be regulated. That said, restoration efforts, such as increasing tree density and basal area, could improve the survival of stands [16], which in turn promotes effective carbon burial [17]. If a large soil carbon pool is sustainably managed, permanence is largely secured. Efforts to reduce emissions from mangrove deforestation and degradation must prioritize protecting soil carbon stocks as they contribute up to 80% of the mangrove blue carbon ecosystem [18].

Here we present a synthesis of carbon stock and flux datasets from 249 mangrove ecosystems in Indonesia, and further derive EFs associated with multiple land management regimes (e.g., aquaculture, degraded mangrove, regenerated mangrove, and undisturbed mangrove). We used a systematic review approach to address the primary question—to what extent does land-use change affect carbon stocks and GHG flux dynamics in Indonesian mangrove ecosystems? Our synthesis and dataset on mangrove EFs will be useful to support future improvements in Forest Reference Emission Level (FREL), as well as to support emerging mangrove restoration projects related to voluntary carbon offset mechanisms.

## Methods

### Literature search

The scope and steps of this study followed the protocol used in a previously-published systematic review assessing carbon stocks and GHG fluxes associated with land-use and land-cover change in mangrove ecosystems globally [19]. In line with our focus on Indonesia, we modified the keyword search strings for this review’s literature search (see Table 1). Literature searches were conducted in two main databases (Scopus and Web of Science), with additional searches conducted through Google Scholar. We used both Google Scholar English and Bahasa Indonesia, collecting the first 50 literature results, in ‘most relevant’ order. Literature searches were conducted several times, with the final search undertaken on 24 May 2021. To maintain the quality of literature data, we included only peer-reviewed publications in our systematic review.

**Table 1:** Keywords used as search strings in the literature database search

Category	Search terms
Populations	mangrove* OR Rhizophora OR coast* AND Indonesia
Interventions	undisturb* OR clear* OR pristine OR intact OR plantation OR log* OR harvest* OR abandoned OR anthrop* OR impact* OR aquaculture* OR aquaculture* OR “land use*” OR “oil palm” OR “shrimp farm*” OR “shrimp pond*” OR “rice cultivation” OR “rice farm*” OR “rice production” OR “rice field*” OR “rice area*” OR “fish farm*” OR “fish pond*” OR mining OR degrad* OR disturb* OR “land cover*” OR “urban development” OR deforest* OR conversion OR rehabilit* OR restor* OR pollut* OR erosion
Comparator	ecosystem OR sediment* OR biomass OR soil* OR NPP OR productivity OR “root product*” OR dynamic* OR litter* OR “dead wood” OR emission* OR stock* OR storage* OR respiration OR efflux OR sequest* OR soil OR forest* OR POC OR DOC OR DIC OR burial
Outcomes	carbon OR methane OR “greenhouse gas*” OR GHG OR flux* OR emission* OR CO2 OR CH4 OR N2O

### Literature screening

We conducted three stages of literature screening, which included title, abstract and full-text screening. The numbers of included and excluded literature are described in the Preferred Reporting Items for Systematic Review and Meta-Analysis (PRISMA) diagram (Table 2). Included studies needed to meet the predefined scope and inclusion criteria of the previous systematic review on the effect of land-use change on mangrove carbon stocks [18]. Specifically, we included only field-based data collection studies from Indonesia that reported carbon stocks (aboveground and belowground biomass, woody debris, and soil carbon pools) as well as carbon fluxes (biomass productivity, GHG fluxes, aquatic carbon fluxes, and soil carbon burial) from either and/or both undisturbed and disturbed mangrove ecosystems. At each stage of the literature screening, we developed semi-structured questions to assess the relevancy of each literature, in terms of the scope of the systematic review.

**Table 2:** The PRISMA systematic review workflow for literature screening, inclusion, and exclusion

Process	Inclusion	Exclusion
Literature identification	n = 163 (Scopus, cut-off date 27 Feb 2020)	
	n = 137 (WoS, cut-off date 27 Feb 2020)	
	n = 45 (Scopus: cut-off date 24 May 2021)	
	n = 44 (WoS: cut-off date 24 May 2021)	
	n = 2 (author contact)	
	n = 691 (all search)	
	↓	
	n = 321 (remaining literatures) ⟶	n = 370 (duplicates)
	↓	
Literature screening	n = 98 (title screening) ⟶	n = 223 (irrelevant literatures)
	↓	
	n = 66 (abstract screening) ⟶	n = 32 (irrelevant literatures)
	↓	
	n = 47 (full text screening) ⟶	n = 19 (irrelevant literatures)
	↓	
Eligibility evaluation	n = 45 (critically appraised) ⟶	n = 2 (irrelevant literatures)
	↓	
Included literature	n = 29 studies were eligible for data extraction and analysis ⟶	n = 16 (irrelevant literatures)

### Critical appraisal and data extraction

We used three main criteria to assess the quality of the datasets presented in included literature. Literature included for data extraction had to meet the following criteria: (a) location and land-use types of the study site(s) are described; (b) carbon stocks and/or GHG fluxes data, obtained from primary field-based collection, are available; (c) rigorous statistical assessment, such as sufficient replication. Literature was excluded from data extraction if not all three criteria were met.

Data extraction focused on the following items: AGBC, BGBC, DOM, SOC, soil CO_2_ efflux, soil CH_4_ efflux, soil N_2_O efflux, and soil carbon burial. We extracted all standard data presentations, such as mean, standard deviation, standard error and sample sizes for each study. If data were presented in figure format and direct reading was not possible, we retrieved the numbers by using WebPlotDigitizer Version 4.0 [20]. All data were compiled into a single database (see Supplementary Data for details, including variables and compilation of extracted data for this review study).

### Data analysis

We summarized datasets by using the descriptive statistical approach for carbon stock pools and flux pathways, across four different land-use types (e.g., aquaculture, degraded mangrove, regenerated mangrove and undisturbed mangrove). The meta-analysis was applied to calculate the effect-size, mean, standard error, and confidence interval of data obtained from multiple studies, before these were summarized according to carbon pool, flux pathways, and land management regime, by using *metafor* R package in R statistics [21].

The percentage of change in biomass and DOM carbon pools, comparing between undisturbed mangrove and disturbed mangrove classes (i.e., aquaculture and degraded mangrove), was calculated using the carbon stock difference approach, while the percentage of change in soil carbon was quantified using a meta-analysis approach (e.g., by comparing with ratio effect size). The meta-analysis was run using OpenMee open-access software for ecology and evolutionary biology meta-analysis [22]. Data that reported carbon stocks and GHG fluxes across regenerated mangroves were not included in the stock change and meta-analysis; these were instead analyzed and presented separately to examine the rate of carbon storage recovery according to revegetation stage (e.g., restoration, rehabilitation).

## Results

### Data availability and distribution

**Fig. 1:**
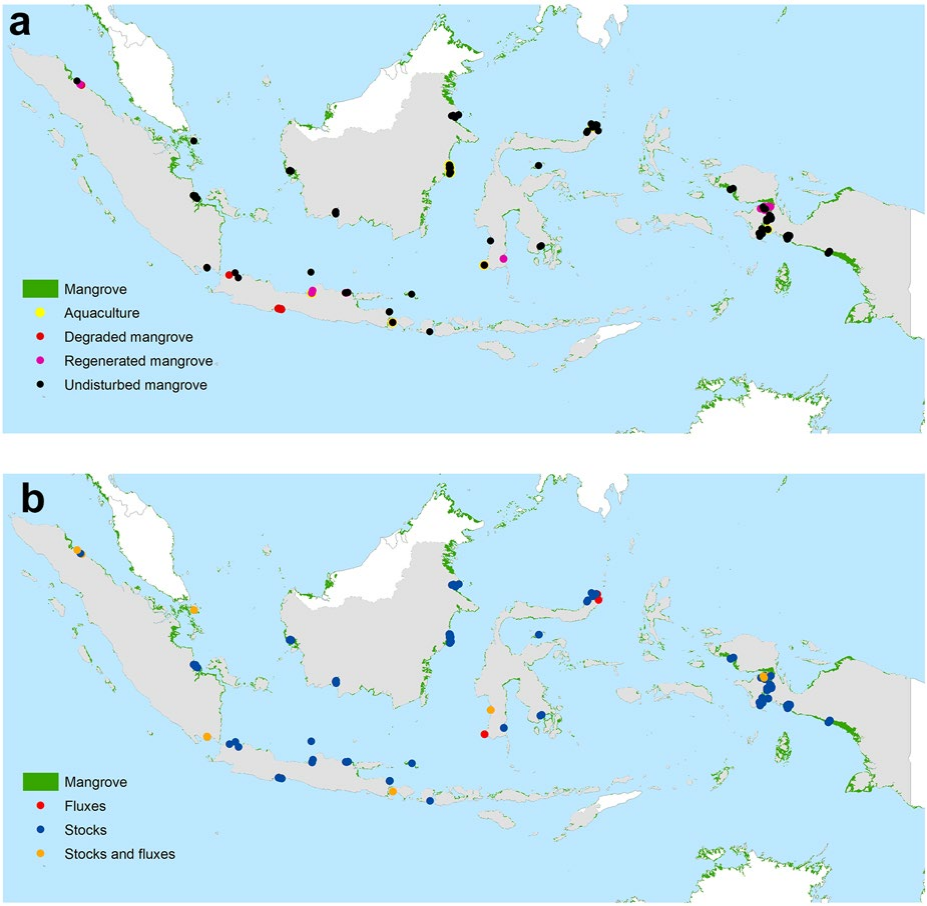
Geographical distribution of 249 studies’ locations in Indonesia (not to scale; the coordinates of the sites are indicated in the individual publication). The top panel shows study locations categorized by land management types, i.e., aquaculture (21 studies), degraded mangrove (18 studies), regenerated mangrove (57 studies), and undisturbed mangrove (153 studies). The bottom panel presents study locations categorized by the type of compiled dataset, i.e., carbon stocks (184 studies), GHG fluxes (37 studies), and carbon stocks and GHG fluxes (28 studies)

With the geographical distribution of our systematic review only focusing on Indonesia (Fig. 1), we compiled 736 relevant data collected across 249 study sites from 29 peer-reviewed publications, to derive EFs in mangrove ecosystems (Table 3). We observed that 85% of the data related to carbon stocks, while just 15% related to GHG and soil carbon fluxes. The records also indicate that publications on this subject have increased significantly in number over the last decade, suggesting a growing interest in the topic by the scientific community, particularly after 2012 (Additional file 1: Fig. S1). Most of the collected data focused on undisturbed mangrove (61%), followed by regenerated mangrove (23%), aquaculture (8%), and degraded mangrove (7%) study sites.

**Table 3:** List of included publications, along with their number of studies across different carbon stock pools and GHG fluxes

No.	Publication^a^	Carbon pool						Soil CO_2_ efflux	Soil CH_4_ efflux	Soil N_2_O efflux	Soil C burial
		AGBC		BGBC		DOM	SOC				
1	Alongi et al. [33]							12	4		
2	Alongi et al. [34]	4	4				4	4	4		
3	Ardhani et al. [35]	3	3		3		5				
4	Arif et al. [36]	5	5				5				
5	Arifanti et al. [27]	18	18				18				
6	Asadi and Pambudi [37]	7	7								
7	Asadi et al. [38]						2				
8	Asadi et al. [39]	1	1								
9	Cameron et al. [28]	13	13				13				
10	Cameron et al. [40]							13	13		
11	Chen et al. [41]							9	9	9	
12	Dharmawan [42]	3	3					3			
13	Hanggara et al. [17]	11	11		10		9				6
14	Hapsari et al. [43]										1
15	Hidayah and Andriyani [44]	1									
16	Jennerjahn [45]						2				2
17	Kangkuso et al. [46 ]	2									
18	Kusumaningtyas et al. [47]	4					4				3
19	Malik et al. [48]	3	3								
20	Murdiyarso et al. [6]	39	39		39		39				
21	Murdiyarso et al. [18]	6	6		3		23				4
22	Nehren and Wicaksono [49]	3	3				3				
23	Rudiastuti et al. [50]	1									
24	Sasmito et al. [15]	49	49		39		49				
25	Sasmito et al. [51]						2				2
26	Sidik and Lovelock [52]							2			
27	Sidik et al. [53]	2	2					2			2
28	Slamet et al. [54]	2	2				2				
29	Weiss et al. [55]						12				
	Total studies	177	169		94		192	45	30	9	20

AGBC, aboveground biomass carbon; BGBC, belowground biomass carbon; DOM, dead organic matter; SOC, soil organic carbon

^a^The full citations are listed in the Supplementary Information

It is interesting to note that field sampling was undertaken across the archipelago (Fig. 1), representing the four main management regimes (e.g., aquaculture, degraded mangrove, regenerated mangrove, and undisturbed mangrove). While carbon stock studies are more widespread, interest in flux studies is growing, allowing us to make more analyses and improve gaps in knowledge and information availability. Widely available carbon stocks data suggests that using a stock difference approach may be more readily available way to derive EFs compared to gain-loss approach [3].

### Carbon stocks and GHG fluxes

**Fig. 2:**
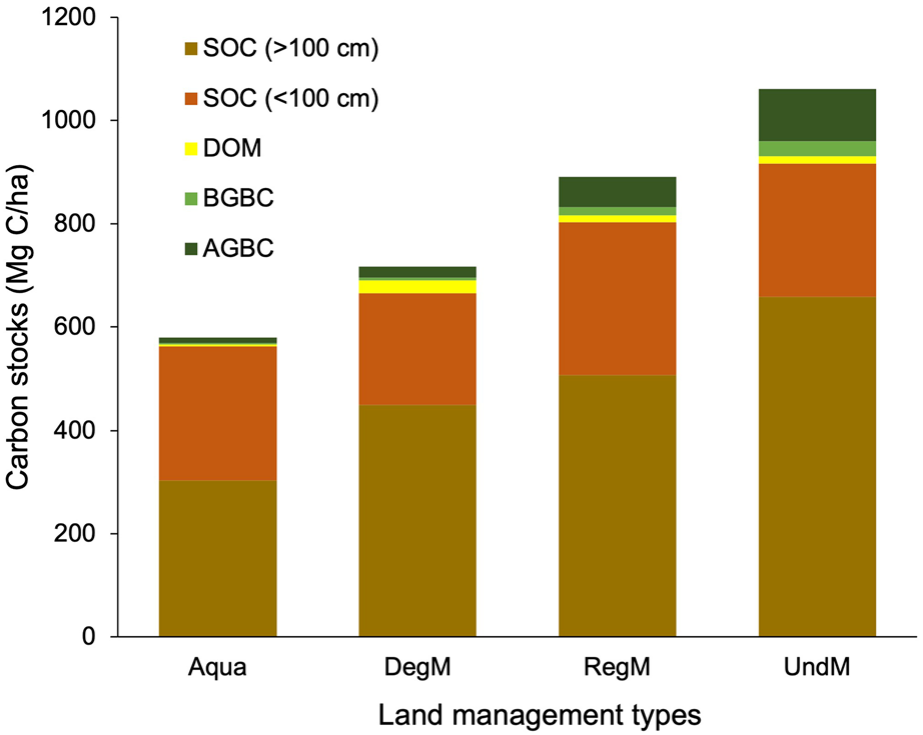
Carbon stocks in various pools across different management regimes (Aqua, aquaculture; DegM, Degraded mangrove; RegM, regenerated mangrove; and UndM, Undisturbed mangrove; AGBC, aboveground biomass carbon; BGBC, belowground biomass carbon; DOM, dead organic matter; SOC, soil organic carbon)

Tier 2 TECS in Indonesian mangrove ecosystems ranged between 579 and 1061 Mg C ha^−1^ depending on their associated land uses (Fig. 2 and Table 4). TECS increased in line with rehabilitation status, with the smallest TECS observed in aquaculture, followed by degraded mangrove, regenerated mangrove and undisturbed mangrove respectively. By contrast, soil carbon stocks in the first 100 cm of depth remained similar across all management regimes, ranging between 216 ± 38 and 296 ± 20 Mg C ha^−1^. Overall, the soil carbon pool contributed the highest proportion of TECS, with 86% found in undisturbed mangroves, 93% and 90% in both degraded and regenerated mangroves, and 97% in aquaculture.

**Table 4:** Carbon stocks in various pools across Indonesian mangrove ecosystems under different management regimes

Carbon stocks	Unit	Mean	n	Standard error	95% CI
*Aquaculture*					
AGBC	Mg C ha^−1^	11.01	10	3.86	2.29–19.73
BGBC	Mg C ha^−1^	2.64	7	1.30	− 0.54 to 5.81
DOM	Mg C ha^−1^	3.39	2	2.72	− 31.15 to 37.93
SOC 0–100 cm	Mg C ha^−1^	259.08	6	90.53	26.37–491.8
SOC 0–300 cm	Mg C ha^−1^	562.36	10	50.20	448.81–675.91
*Degraded mangrove*					
AGBC	Mg C ha^−1^	20.98	8	6.05	6.68–35.28
BGBC	Mg C ha^−1^	6.01	6	1.43	2.34–9.67
DOM	Mg C ha^−1^	24.34	7	6.67	8.03–40.65
SOC 0–100 cm	Mg C ha^−1^	215.66	13	38.07	132.7–298.62
SOC 0–300 cm	Mg C ha^−1^	665.59	6	132.49	325.02–1006.15
*Regenerated mangrove*					
AGBC	Mg C ha^−1^	58.06	31	8.17	41.38–74.75
BGBC	Mg C ha^−1^	15.80	26	3.77	8.04–23.55
DOM	Mg C ha^−1^	13.49	18	2.52	8.18–18.81
SOC 0–100 cm	Mg C ha^−1^	296.41	27	20.11	255.07–337.75
SOC 0–300 cm	Mg C ha^−1^	803.03	15	48.76	698.45–907.61
*Undisturbed mangrove*					
AGBC	Mg C ha^−1^	101.67	114	4.79	92.18–111.16
BGBC	Mg C ha^−1^	28.70	98	1.65	25.42–31.98
DOM	Mg C ha^−1^	14.47	63	1.22	12.03–16.9
SOC 0–100 cm	Mg C ha^−1^	258.44	34	32.40	192.53–324.36
SOC 0–300 cm	Mg C ha^−1^	916.42	75	47.60	821.57–1011.28

The plots of mean, standard error, and confidence interval for all carbon pools calculated using a random-effects model are shown in Additional file 1: Figs. S2–S22

AGBC, aboveground biomass carbon; BGBC, belowground biomass carbon; DOM, dead organic matter; SOC, soil organic carbon; n, number of study

Table 5 shows that the greatest soil CO_2_ efflux was observed in aquaculture—as much as 23.8 ± 7.6 Mg CO_2_ ha^−1^ yr^−1^—followed by regenerated and undisturbed mangroves with 13.5 ± 4.5 and 7.9 ± 1.4 Mg CO_2_ ha^−1^ yr^−1^, respectively. Degraded mangrove generated the largest soil CH_4_ effluxes (4.2 ± 1.5 Mg CO_2_-eq ha^−1^ yr^−1^), followed by aquaculture (2.0 ± 0.7 Mg CO_2_-eq ha^−1^ yr^−1^) and undisturbed mangrove sites (1.0 ± 0.7 Mg CO_2_-eq ha^−1^ yr^−1^). By contrast, the largest mean for soil carbon burial occurred in undisturbed mangrove (3.2 ± 2.2 Mg C ha^−1^ yr^−1^) followed by regenerated mangrove (1.7 ± 0.3 Mg C ha^−1^ yr^−1^) and degraded mangrove (1.2 ± 0.4 Mg C ha^−1^ yr^−1^).

**Table 5:** Greenhouse gas fluxes and soil carbon burial across Indonesian mangrove ecosystems under different management regimes

Carbon fluxes	Unit	Mean	n	Standard error	95% CI
*Aquaculture*					
Soil CO_2_ effluxes	Mg CO_2_ ha^−1^ yr^−1^	23.81	30	1.40	− 0.40 to 48.02
Soil CH_4_ effluxes	Mg CO_2_-eq ha^−1^ yr^−1^	2.02	20	0.68	− 1.11 to 5.16
Soil N_2_O effluxes	kg CO_2_-eq ha^−1^ yr^−1^	NA	9	0.16	NA
Soil carbon burial	Mg C ha^−1^ yr^−1^	NA	7	2.24	NA
*Degraded mangrove*					
Soil CO_2_ effluxes	Mg CO_2_ ha^−1^ yr^−1^	NA	4	7.61	–
Soil CH_4_ effluxes	Mg CO_2_-eq ha^−1^ yr^−1^	4.18	3	0.73	− 0.63 to 8.99
Soil N_2_O effluxes	kg CO_2_-eq ha^−1^ yr^−1^	NA	NA	NA	–
Soil carbon burial	Mg C ha^−1^ yr^−1^	1.22	NA	NA	0.39–2.05
*Regenerated mangrove*					
Soil CO_2_ effluxes	Mg CO_2_ ha^−1^ yr^−1^	13.49	NA	NA	2.39–24.6
Soil CH_4_ effluxes	Mg CO_2_-eq ha^−1^ yr^−1^	NA	4	1.51	–
Soil N_2_O effluxes	kg CO_2_-eq ha^−1^ yr^−1^	NA	NA	NA	–
Soil carbon burial	Mg C ha^−1^ yr^−1^	1.67	8	0.35	0.87–2.46
*Undisturbed mangrove*					
Soil CO_2_ effluxes	Mg CO_2_ ha^−1^ yr^−1^	7.87	7	4.54	5.00–10.74
Soil CH_4_ effluxes	Mg CO_2_-eq ha^−1^ yr^−1^	0.98	NA	NA	− 0.44 to 2.4
Soil N_2_O effluxes	kg CO_2_-eq ha^−1^ yr^−1^	− 0.12	NA	NA	− 0.48 to 0.25
Soil carbon burial	Mg C ha^−1^ yr^−1^	3.20	5	0.29	− 2.28 to 8.69

The plots of mean, standard error, and confidence interval for all GHG fluxes calculated using a random-effects model are shown in Additional file 1: Figs. S23–S31

### Change of carbon stocks and GHG fluxes following land-use change

**Fig. 3:**
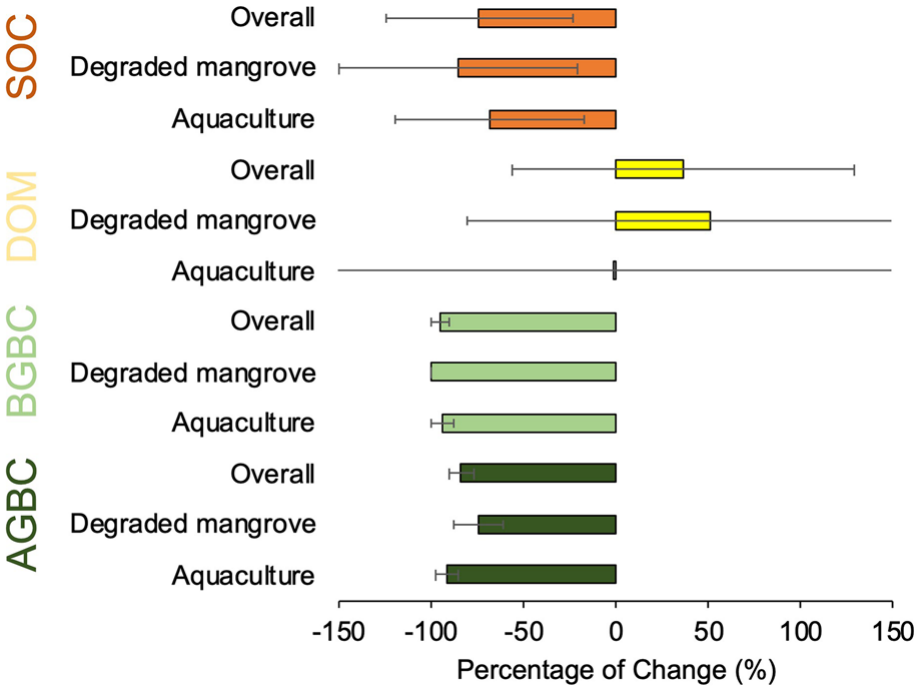
Carbon stock loss and gain (in per cent) following two major land-use changes (aquaculture and mangrove degradation) across the main four carbon pools. AGBC, aboveground biomass carbon; BGBC, belowground biomass carbon; DOM, dead organic matter; SOC, soil organic carbon

We found that land-use change overall generated substantial TECS loss, relative to the undisturbed mangrove baseline (Fig. 3). By using paired datasets of undisturbed mangrove and land-use change impacted sites, however, we further observed that the degree of carbon stock loss within each carbon pool varied depending on the land-use type or management regime (Fig. 3). TECS losses were approximately 64% and 52%, due to aquaculture and degradation respectively. Biomass carbon stocks experienced the greatest loss following land-use change, between 84 and 95%, while soil carbon pool loss was 74%. By contrast, DOM carbon stocks were 36% greater when the area was impacted by land-use change, compared to undisturbed mangroves. This indicates that woody debris may have substantially accumulated due to disturbance regimes.

In the context of carbon gains and losses and GHG fluxes, Table 3 shows that soil CO_2_ fluxes in land-use impacted mangroves were between 71 and 202% greater than those seen in undisturbed mangroves. A similar increase was seen in soil CH_4_ effluxes in mangrove ecosystems following land-use change (between 106 and 326%). The carbon burial dataset also suggests a substantial difference between undisturbed and degraded mangroves. When mangroves were degraded, approximately 48–62% of carbon burial rates were reduced.

**Fig. 4:**
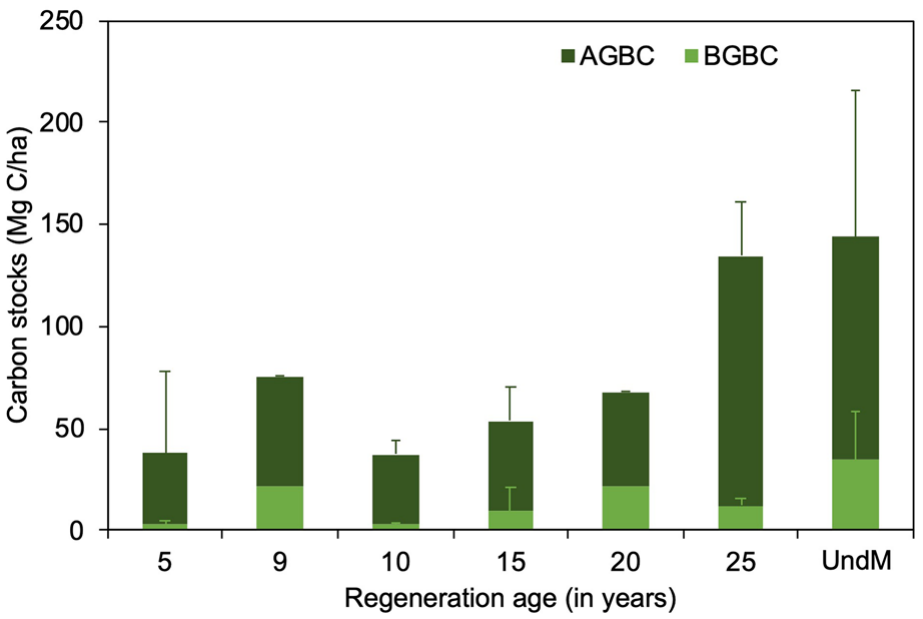
Aboveground and belowground biomass carbon stock recovery according to mangrove regeneration age. UndM denotes undisturbed mangroves

Mangrove regeneration or rehabilitation may not be able to fully recover the loss of TECS, which may have taken place in a relatively short period. Soil carbon stocks in land-use change impacted mangroves were 12–39% lower than in undisturbed mangroves. According to the biomass dataset, however, recovery did occur in aboveground and belowground biomass carbon stocks (Fig. 4). In fact, biomass carbon can be considered to be fully recovered after 25 years of regeneration, when compared with undisturbed mangrove.

## Discussion

### Tier 2 emissions factors for land-use change in mangroves

Our study provides a synthesis of carbon stocks and fluxes from 249 mangrove ecosystems in Indonesia and therefore, these datasets are suitable and useful to support the development of Tier 2 EFs. On comparing the baseline EFs between the Tier 1 default in the 2013 IPCC Wetlands Supplement [2] and our findings, we found that the default value for the AGBC pools for tropical wet mangrove (86 Mg ha^−1^) is lower than that found in this study (102 Mg ha^−1^). Meanwhile, the value for top 1 m soil carbon in this study (296 Mg ha^−1^) was found to be lower than the IPCC default value of 471 Mg ha^−1^.

It is worth noting, however, that the population of the datasets differs substantially. The default value for the AGBC was calculated using 49 studies, while this meta-analysis used 114 studies only for Indonesia. For soil carbon, sample size for the default value was 43 (presumably from all climatic zones and multiple countries), while for the Indonesian value it was 27. This suggests that the proposed Tier 2 AGBC is highly reliable and indicates the potential to maintain long-term conservation management efforts rather than extensive rehabilitation and restoration efforts, given the evidence that mangrove plantations are inadequate to recover long-term carbon capture and storage, and the result of regeneration may not resemble the structure and species diversity of undisturbed mangrove [23–25].

The Tier 2 soil carbon data proposed here were all collected from the same climatic zone (tropical-wet), and were impacted by very dynamic land-use changes, including highly destructive soil excavation during aquaculture development. The deeper layer (i.e., up to 300 cm) soil carbon dataset reached an optimum baseline of 916 Mg ha^−1^. This suggests the potential permanence that blue carbon mangrove projects have; as well as the safeguarding they offer to national carbon accounting under the existing MRV system, when it comes to further leakage or emission displacement in wetland ecosystems. With the large soil carbon dataset that exists from 300 cm deep cores (n = 75), it has been proposed that a deeper layer baseline (rather than the standard 1-m depth) could be used in areas where aquaculture is rampant [6].

### Stock-difference and gain–loss approaches

The majority of currently available publications reported data on carbon stocks, rather than GHG fluxes, in Indonesian mangrove ecosystems. This provides a greater opportunity to apply a stock-difference rather than a gain–loss approach for the Indonesian National Greenhouse Gas Inventory. This stock-difference approach has been proven to produce considerably smaller estimates of uncertainty in estimating forest carbon emissions for aboveground biomass pools [26].

A comparison of sites suggests that carbon stock losses due to aquaculture conversion lead to different emissions factors. For example, aquaculture reduced carbon stocks at the largest scale (75%) when compared to forest logging (27%) and regenerated forest (17%) [15]. Aquaculture conversion in Mahakam and Tanakeke, in the island of Kalimantan and Sulawesi, generated lower carbon stocks loss at just 48% and 36% respectively [27, 28]. These findings imply that emissions factors, following mangrove conversion into other land-uses, are unique depending on geographical location and thus on site-specific hydrogeomorphic characteristics, as well as the type and duration of new land use that has replaced mangroves. It is also possible that local practices and culture determine how fish and shrimp ponds are established (e.g., different soil excavation depth) and managed, hence, the resulting GHG emissions.

### Future refinement

While Tier 1 default EFs mostly provide the absolute mean values for carbon stocks and fluxes, their accuracy, and whether these EFs are consistent enough to be used for multiple key categories, land representations and geographical locations, remains unclear. Aquacultural land carbon stocks, for example, can differ between countries depending on the initial carbon stocks prior to conversion as well as soil excavation depth. Following carbon stock and flux change analysis previously conducted on a global level [29], in this study we also calculated the relative change of carbon stocks for each pool (provided as percentage of change), in response to land-use change specifically for Indonesia (i.e., degraded mangrove and aquaculture, Fig. 3). These relative changes are particularly useful to understand carbon loss impacts between types of land use change, if countries or projects have calculated carbon stocks based upon undisturbed mangrove alone. Relative carbon stock changes can also be used to support more advanced analysis, such as the modelling of emissions and removals projections [30].

Further refinement of the soil carbon data is required, so as to improve consistency when calculating carbon emissions by using stocks different approach. In the studies and data analyzed here, soil carbon stocks were obtained from diverse soil depths; for example, soil carbon in Mahakam was obtained from a depth of 300 cm, while in other areas, sampling was undertaken from depths of up to 100 cm only (see Supplementary Data). The depth of soil organic matter is the main factor controlling variation in soil carbon stocks between sites and particularly hydrogeomorphic settings [15]. As seen in mangrove carbon stock assessments elsewhere, the soil carbon pool contributes the most (> 50%) toward TECS. As deeper soil coring also implies cost effectiveness, the modelling of deeper layer carbon stocks value should be pursued, since this will introduce fewer uncertainties, compared with estimating soil carbon based upon AGBC, as is widely-practiced currently [31]. The inclusion of the DOM carbon pool in carbon emissions and removals reporting may also improve accuracy. Despite this carbon pool only contributing up to 1.4% of TECS in undisturbed mangrove (Fig. 2), it is often neglected and reported rarely by most studies [14]. Changes in the DOM carbon pool are reported to be significant, particularly when mangroves experience direct biomass removals, through logging, for example [15, 17]. The inclusion of DOM will certainly improve accuracy in emissions and removals reporting, particularly where forest management is applied for wood resource extraction. The highest DOM found in degraded mangrove of 24.34 ± 6.67 Mg ha^−1^ is comparable with those found in Kenya of 29.92 ± 36.72 Mg ha^−1^ [32]^.^

### Implications for Indonesia’s national greenhouse gas inventory

The results of this study reflect TECS for Indonesia’s blue carbon mangrove ecosystems, categorized into four site conditions—undisturbed mangroves, regenerated mangroves, degraded mangroves and aquaculture. These allow us to understand the factors driving carbon stock changes. As recommended by IPCC best practice, country-specific EFs should be developed for each ecosystem, in its various conditions. Our results offer the opportunity to improve the accuracy and reduce the uncertainty of country-scale accounting for emissions associated with land-use change in mangrove ecosystems.

The EFs derived in this study are essential, not only for the estimation of GHG emissions, but also for evaluating the progress made by mitigation programs in reducing emissions. Application of these EFs in Indonesia’s GHG emission accounting under both jurisdictional and project carbon financing schemes should be straightforward. The results can also be adopted by the IPCC emission factor database, allowing countries to use EFs that are suitable for their specific mangrove situation, thus helping to improve the quality of GHG inventories in a cost-effective way.

## Conclusions

The derived Tier 2 EFs for mangrove blue carbon ecosystems in Indonesia were derived from a systematic review of 29 peer-reviewed publications are geographically well distributed across the archipelago. Following IPCC Guidelines, the EFs were separated into carbon pools, i.e., aboveground, belowground, dead organic matter and soil. They are readily useable for Indonesia’s National Greenhouse Gas Inventory and the reporting of climate change mitigation measures. The ecosystems are categorized by management regime: aquaculture, degraded mangroves, regenerated mangroves, and undisturbed mangroves.

Using deeper layer (e.g., 300 cm) soil carbon storage values would compensate for the underestimation in carbon losses from coastal ecosystems where aquaculture is widely practised. From a project perspective, this deeper layer data could secure permanence or buffer potential leakages; while from a national GHG accounting perspective, it also provides a safeguard in the MRV system.

## Supplementary Information

**Additional file 1.** Trends of publications on mangrove blue carbon in Indonesia and random effects model results by carbon pools and land management types.

## Data Availability

The dataset presented in this paper is available at the following link: 10.6084/m9.figshare.23660085.

## References

[1] UNFCCC. Handbook on measurement, reporting and verification for developing country parties*.* Bonn: United Nations Framework Convention on Climate Change Secretariat, Bonn; 2014.

[2] Hiraishi T, Krug T, Tanabe K, Srivastava N, Baasansuren J, Fukuda M, Troxler TG. (eds). Methodology report. In: IPCC. 2013 Supplement to the 2006 IPCC guidelines for national greenhouse gas inventories: wetlands. Switzerland: IPCC; 2014

[3] IPCC. Guidelines for national greenhouse gas inventories—volume 4: Agriculture, land use and forestry (GL-AFOLU). IPCC. 2006. http://www.ipcc-nggip.iges.or.jp. Accessed 24 May 2021.

[4] Kelleway JJ (2020). A national approach to greenhouse gas abatement through blue carbon management. Glob Environ Change.

[5] FAO. The world’s mangroves 1980–2005: a thematic study prepared in the framework of the global forest resources assessment 2005. FAO: Rome; 2007.

[6] Murdiyarso D (2015). The potential of Indonesian mangrove forests for global climate change mitigation. Nat Clim Change.

[7] Griscom BW (2020). National mitigation potential from natural climate solutions in the tropics. Phil Trans R Soc B.

[8] Nikalje GC, Bhaskar SD, Yadav K, Penna S, Hasanuzzaman M, Nahar K, Öztürk M (2019). Halophytes: prospective plants for future. Ecophysiology, abiotic stress responses and utilization of halophytes.

[9] Xiong Y, Liu X, Guan W, Liao B, Chen Y, Li M, Zhong C (2016). Fine root functional group based estimates of fine root production and turnover rate in natural mangrove forests. Plant Soil.

[10] Nellemann C, et al. editors. Blue carbon: a rapid response assessment. 2009. www.grida.no. Accessed 24 May 2021.

[11] Mcleod E (2011). A blueprint for blue carbon: toward an improved understanding of the role of vegetated coastal habitats in sequestering CO_2_. Front Ecol Environ.

[12] Donato DC, Kauffman JB, Murdiyarso D, Kurnianto S, Stidham M, Kanninen M (2011). Mangroves among the most carbon-rich forests in the tropics. Nat Geo Sci.

[13] Saintilan N, Rogers K, Mazumder D, Woodroffe C (2013). Allochthonous and autochthonous contributions to carbon accumulation and carbon store in southeastern Australian coastal wetlands. Estuar Coast Shelf Sci.

[14] Kauffman JB (2020). Total ecosystem carbon stocks of mangroves across broad global environmental and physical gradients. Ecol Monogr.

[15] Sasmito SD (2020). Mangrove blue carbon stocks and dynamics are controlled by hydrogeomorphic settings and land-use change. Glob Change Biol.

[16] Kathiresan K, Saravanakumar K, Anburaj R (2016). A simple method for assessing mangrove forest based on young plants and sesarmid crab holes. Reg Stud Mar Sci.

[17] Hanggara BB, Murdiyarso D, Ginting YRS, Widha YL, Panjaitan GY, Lubis AA (2021). Effects of diverse mangrove management practices on forest structure, carbon dynamics and sedimentation in North Sumatra, Indonesia. Estuar Coast Shelf Sci.

[18] Murdiyarso D, Sasmito SD, Sillanpää M, MacKenzie R, Gaveau D (2021). Mangrove selective logging sustains biomass carbon recovery, soil carbon, and sediment. Sci Rep.

[19] Sasmito SD, Taillardat P, Clendenning J, Friess DA, Murdiyarso D, Hutley LB, Carbon stocks and fluxes associated with land-use and land-cover change in mangrove ecosystems: a systematic review protocol. Working Paper No. 211. Indonesia: CIFOR: 2016.

[20] Rohatgi A, Rehberg S, Stanojevic Z. WebPlotDigitizer: Version 4.0. Computer Software. 2018.

[21] Viechtbauer W (2010). Conducting meta-analyses in R with the metafor package. J Stat Softw.

[22] Wallace BC, Lajeunesse MJ, Dietz G, Dahabreh IJ, Trikalinos TA, Schmid CH, Gurevitch J (2017). Open MEE: intuitive, open-source software for meta-analysis in ecology and evolutionary biology. Methods Ecol Evol.

[23] Alongi DM (2011). Carbon payments for mangrove conservation: ecosystem constraints and uncertainties of sequestration potential. Environ Sci Pol.

[24] Irving AD, Connell SD, Russell BD (2011). Restoring coastal plants to improve global carbon storage: reaping what we sow. PLoS ONE.

[25] Sidik F, Supriyanto B, Krisnawati H, Muttaqin MZ (2018). Mangrove conservation for climate change mitigation in Indonesia. WIREs Clim Change.

[26] McRoberts RE, Naesset E, Gobakken T (2018). Comparing the stock-change and gain-loss approaches for estimating forest carbon emissions for the aboveground biomass pool. Can J For Res.

[27] Arifanti VB, Kauffman JB, Hadriyanto D, Murdiyarso D, Diana R (2019). Carbon dynamics and land use carbon footprints in mangrove-converted aquaculture: the case of the Mahakam Delta, Indonesia. For Ecol Manag.

[28] Cameron C, Hutley LB, Friess DA, Brown B (2019). Community structure dynamics and carbon stock change of rehabilitated mangrove forests in Sulawesi, Indonesia. Ecol Appl.

[29] Sasmito SD, Taillardat P, Clendenning JN, Cameron C, Friess DA, Murdiyarso D, Hutley LB (2019). Effect of land-use and land-cover change on mangrove blue carbon: a systematic review. Glob Change Biol.

[30] Adame MF (2021). Future carbon emissions from global mangrove forest loss. Glob Change Biol.

[31] Bukoski JJ, Broadhead JS, Donato DC, Murdiyarso D, Gregoire TG (2017). The use of mixed effects models for obtaining low-cost ecosystem carbon stock estimates in mangroves of the Asia-Pacific. PLoS ONE.

[32] Mugi LM, Kiss D, Kairo JG, Huxham MR (2022). Stocks and productivity of dead wood in mangrove forests: a systematic literature review. Front For Glob Change.

[33] Alongi DM. Mangrove forests: resilience, protection from tsunamis, and responses to global climate change. Estuar Coast Shelf Sci. 2008;76:1–13. 10.1016/j.ecss.2007.08.024.

[34] Alongi DM, Murdiyarso D, Fourqurean JW, Kauffman JB, Hutahaean A, Crooks S, et al. Indonesia’s blue carbon: a globally significant and vulnerable sink for seagrass and mangrove carbon. Wetlands Ecol Manage [Internet]. 2016;24:3–13. 10.1007/s11273-015-9446-y.

[35] Ardhani TSP, Murdiyarso D, Kusmana C. Effects of permeable barriers on total ecosystem carbon stocks of mangrove forests and abandoned ponds in Demak District, Central Java, Indonesia. Biodiversitas. 2020;21(11).10.13057/biodiv/d211134.

[36] Arif AM, Guntur G, Ricky AB, Novianti P, Andik I. Mangrove ecosystem C-stocks of Lamongan, Indonesia and its correlation with forest age. Res J Chem Environ. 2017;10.

[37] Asadi MA, Pambudi GS. Diversity and biomass of mangrove forest within Baluran National Park, Indonesia. AACL Bioflux. 2020;13:19–27.

[38] Asadi MA, Yona D, Fikri MZ. Comparing carbon in sediment of primary and artificially generated mangrove forests. Disaster Advances. 2018;11:18–26.

[39] Asadi MA, Yona D, Saputro SE. Species Diversity, Biomass, and Carbon Stock Assessments of Mangrove Forest in Labuhan, Indonesia. IOP Conference Series: Earth and Environmental Science. Institute of Physics Publishing; 2018.

[40] Cameron C, Hutley LB, Friess DA, Munksgaard NC. Hydroperiod, soil moisture and bioturbation are critical drivers of greenhouse gas fluxes and vary as a function of land use change in mangroves of Sulawesi, Indonesia. Sci Total Environ. 2019;654:365–77. 10.1016/j.scitotenv.2018.11.092 30447576

[41] Chen GC, Ulumuddin YI, Pramudji S, Chen SY, Chen B, Ye Y, et al. Rich soil carbon and nitrogen but low atmospheric greenhouse gas fluxes from North Sulawesi mangrove swamps in Indonesia. Sci Total Environ. 2014;487:91–6. 10.1016/j.scitotenv.2014.03.140 24784732

[42] Dharmawan IWE. CO_2_ dynamics on three habitats of mangrove ecosystem in Bintan Island, Indonesia. IOP Conference Series: Earth and Environmental Science. Institute of Physics Publishing; 2018.

[43] Hapsari KA, Jennerjahn TC, Lukas MC, Karius V, Behling H. Intertwined effects of climate and land use change on environmental dynamics and carbon accumulation in a mangrove‐fringed coastal lagoon in Java, Indonesia. Glob Change Biol. 2020;26:1414–31. https://onlinelibrary.wiley.com/doi/10.1111/gcb.14926 10.1111/gcb.14926 31820533

[44] Hidayah Z, Andriyani L. Carbon Stock Analysis of Mangrove Ecosystems in Paliat Island Sumenep East Java. IOP Conference Series: Earth and Environmental Science. Institute of Physics Publishing; 2019.

[45] Jennerjahn TC. Relevance and magnitude of “Blue Carbon” storage in mangrove sediments: Carbon accumulation rates vs. stocks, sources vs. sinks. Estuar Coast Shelf Sci. 2020;247:107027.

[46] Kangkuso A, Sharma S, Jamili J, Septiana A, Sahidin I, Rianse U, et al. Trends in allometric models and aboveground biomass of family Rhizophoraceae mangroves in the Coral Triangle ecoregion, Southeast Sulawesi, Indonesia. J Sustain Forestry. 2018;37:691–711. 10.1080/10549811.2018.1453843.

[47] Kusumaningtyas MA, Hutahaean AA, Fischer HW, Pérez-Mayo M, Ransby D, Jennerjahn TC. Variability in the organic carbon stocks, sources, and accumulation rates of Indonesian mangrove ecosystems. Estuar Coast Shelf Sci. 2019;218:310–23.

[48] Malik A, Jalil AR, Arifuddin A, Syahmuddin A. Biomass carbon stocks in the mangrove rehabilitated area of Sinjai district, South Sulawesi, Indonesia. Geogr Environ Sustain 2020;13:32–8.

[49] Nehren U, Wicaksono P. Mapping soil carbon stocks in an oceanic mangrove ecosystem in Karimunjawa Islands, Indonesia. Estuar Coast Shelf Sci. 2018;214:185–93.

[50] Rudiastuti AW, Yuwono DM, Hartini S. Mangrove Mapping Using SPOT 6 at East Lombok Indonesia. IOP Conference Series: Earth and Environmental Science. Institute of Physics Publishing; 2018.

[51] Sasmito SD, Kuzyakov Y, Lubis AA, Murdiyarso D, Hutley LB, Bachri S, et al. Organic carbon burial and sources in soils of coastal mudflat and mangrove ecosystems. CATENA. 2020;187:104414.

[52] Sidik F, Lovelock CE. CO_2_ Efflux from Shrimp Ponds in Indonesia. PLoS ONE. 2013;8:e66329. 10.1371/journal.pone.0066329. 10.1371/journal.pone.0066329 PMC3674011 23755306

[53] Sidik F, Fernanda Adame M, Lovelock CE. Carbon sequestration and fluxes of restored mangroves in abandoned aquaculture ponds. J Indian Ocean Region. 2019;15:177–92. 10.1080/19480881.2019.1605659.

[54] Slamet NS, Dargusch P, Aziz AA, Wadley D. Mangrove vulnerability and potential carbon stock loss from land reclamation in Jakarta Bay, Indonesia. Ocean Coastal Manag. 2020;195.

[55] Weiss C, Weiss J, Boy J, Iskandar I, Mikutta R, Guggenberger G. Soil organic carbon stocks in estuarine and marine mangrove ecosystems are driven by nutrient colimitation of P and N. Ecol Evol. 2016;6:5043–56. 10.1002/ece3.2258. 10.1002/ece3.2258 PMC4979726 27547332

