# Supplementary material for: Deriving emission factors for mangrove blue carbon ecosystem in Indonesia
Source: Carbon Balance Manag. 2023 Jul 13;18:12. doi: 10.1186/s13021-023-00233-1 (PMC10339514; doi:10.1186/s13021-023-00233-1)
Supplement: Supplementary file 1 — Additional file 1. Trends of publications on mangrove blue carbon in Indonesia and random effects model results by carbon pools and land management types. [file 13021_2023_233_MOESM1_ESM.docx]

Supplementary Information

**Deriving emission factors for mangrove blue carbon ecosystem in Indonesia**


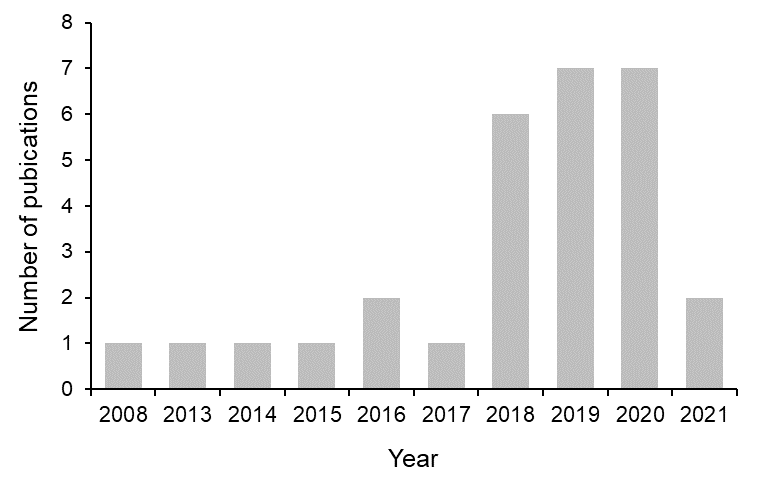


Figure S1. Number of publications included in this study by year


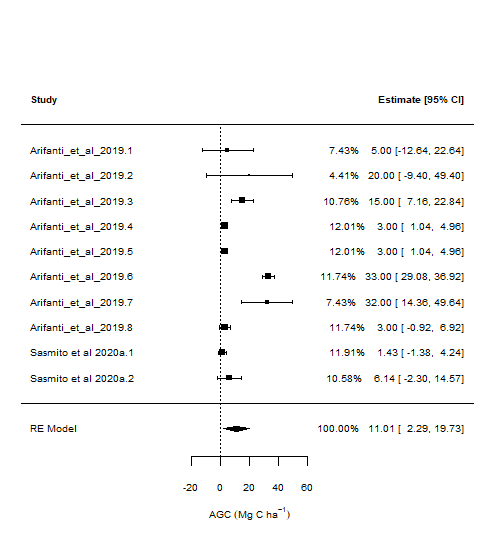


Figure S2. Random effects model results of aboveground biomass carbon stocks in the aquaculture


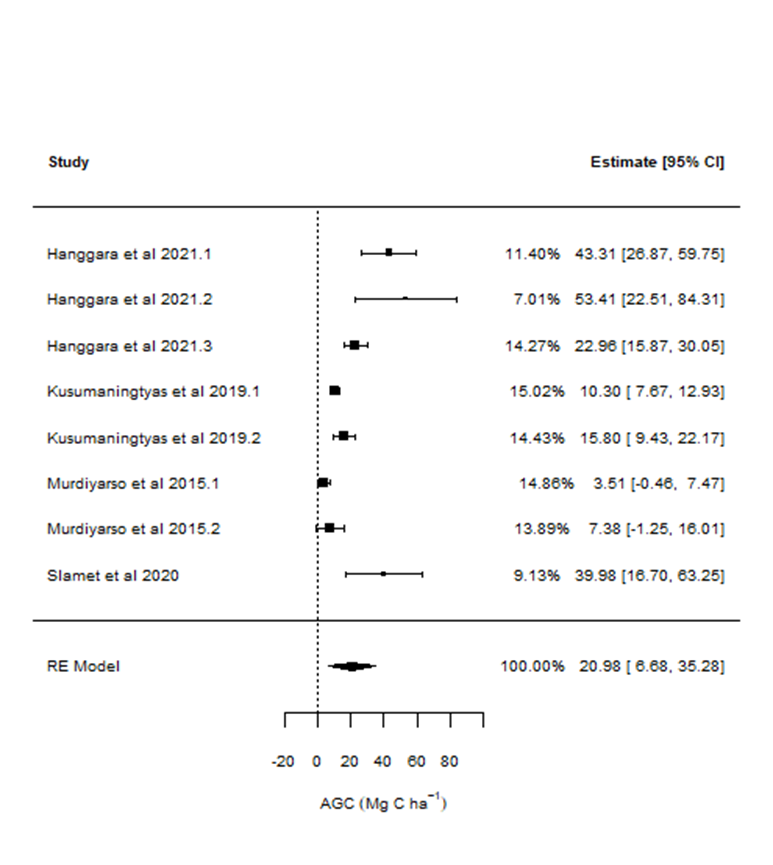


Figure S3. Random effects model results of aboveground biomass carbon stocks in the degraded mangrove


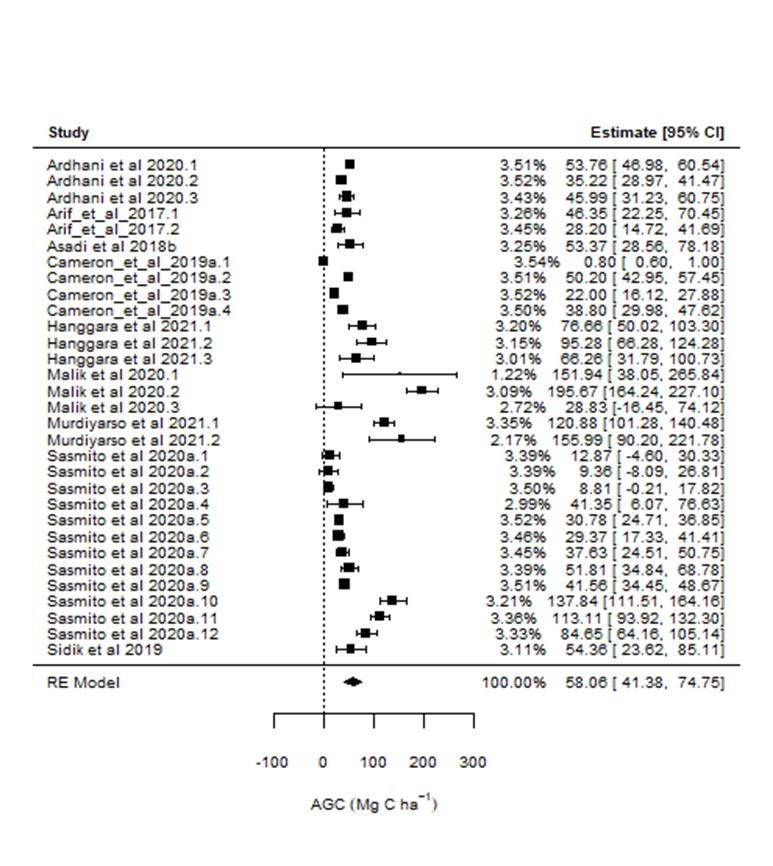


Figure S4. Random effects model results of aboveground biomass carbon stocks in the regenerated mangrove


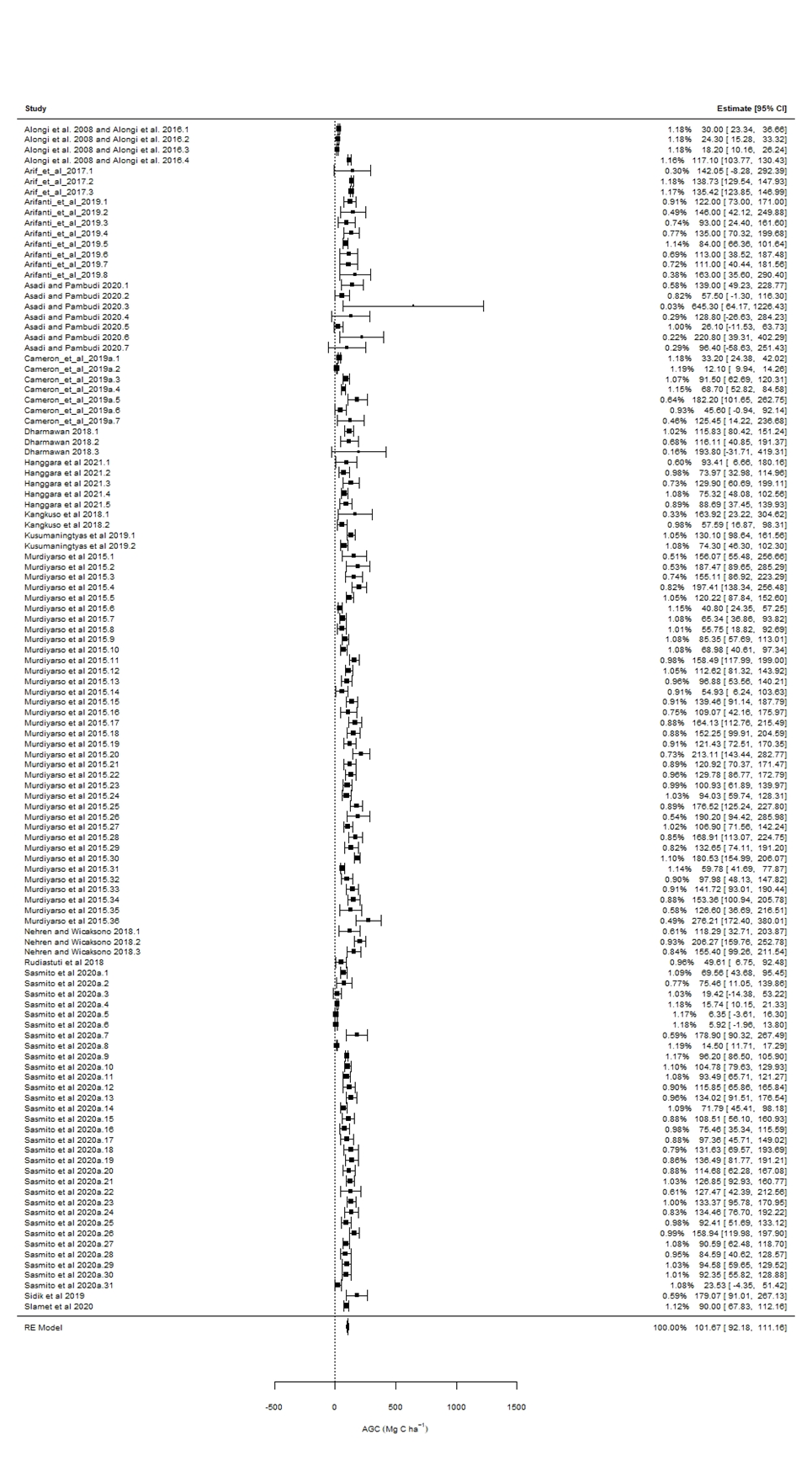


Figure S5. Random effects model results of aboveground biomass carbon stocks in the undisturbed mangrove


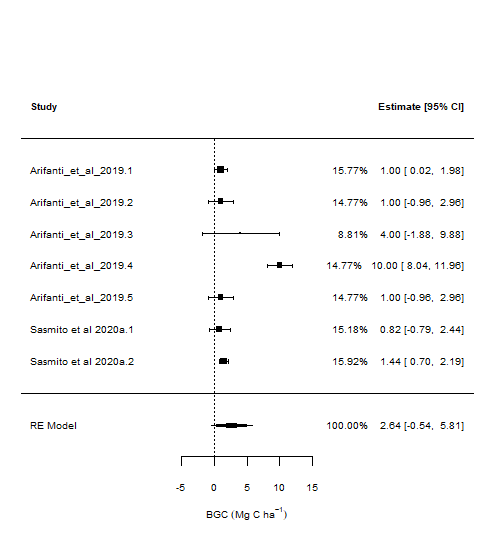


Figure S6. Random effects model results of belowground biomass carbon stocks in the aquaculture


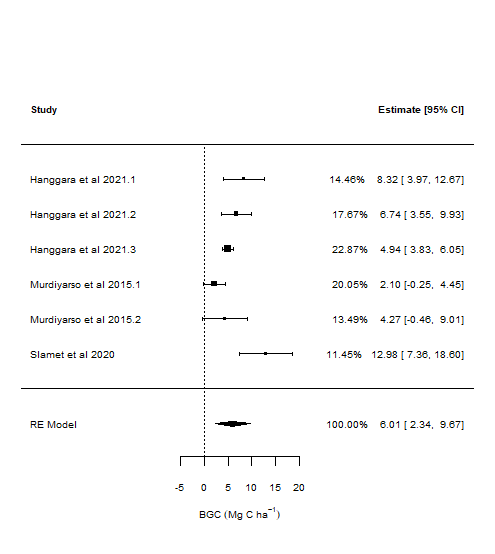


Figure S7. Random effects model results of belowground biomass carbon stocks in the degraded mangrove


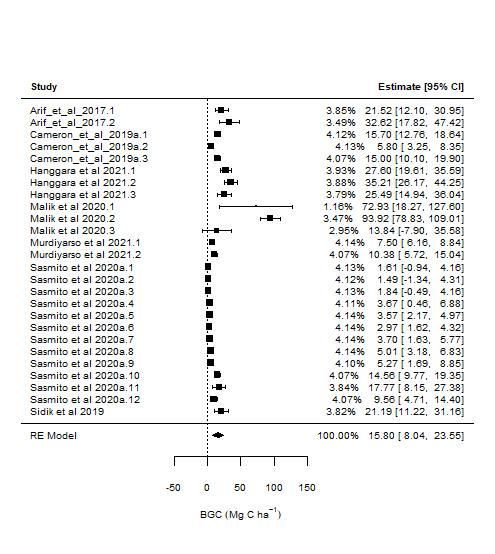


Figure S8. Random effects model results of belowground biomass carbon stocks in the regenerated mangrove


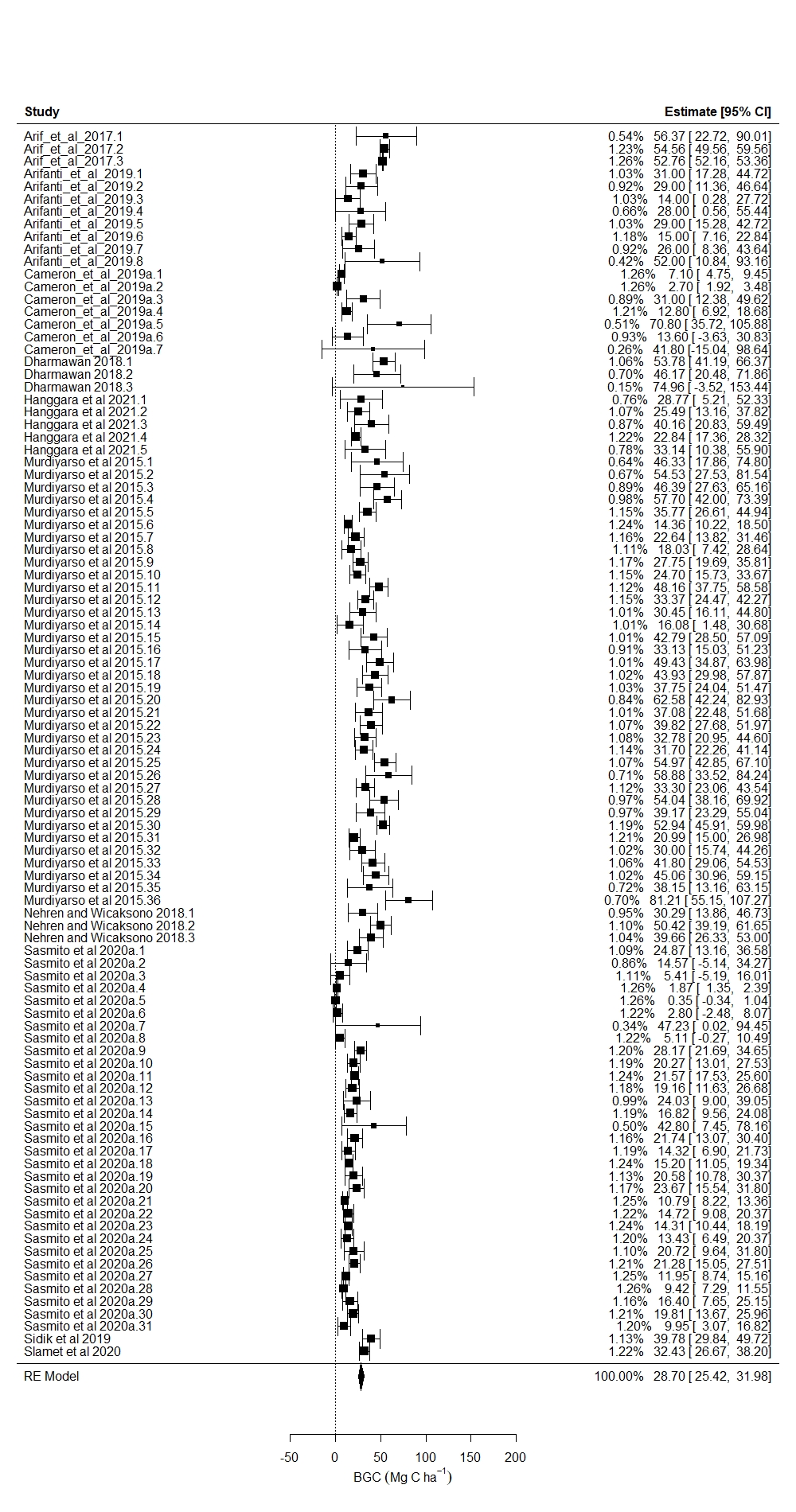


Figure S9. Random effects model results of belowground biomass carbon stocks in the undisturbed mangrove


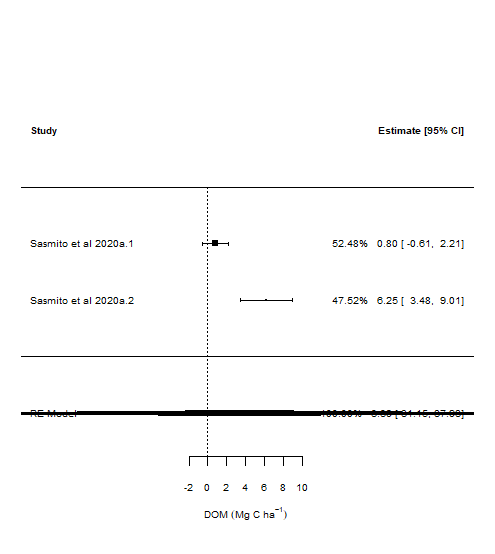


Figure S10. Random effects model results of dead organic matter carbon stocks in the aquaculture


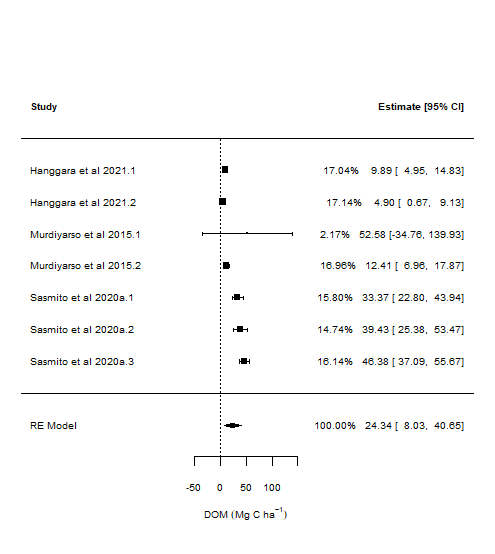


Figure S11. Random effects model results of dead organic matter carbon stocks in the degraded mangrove


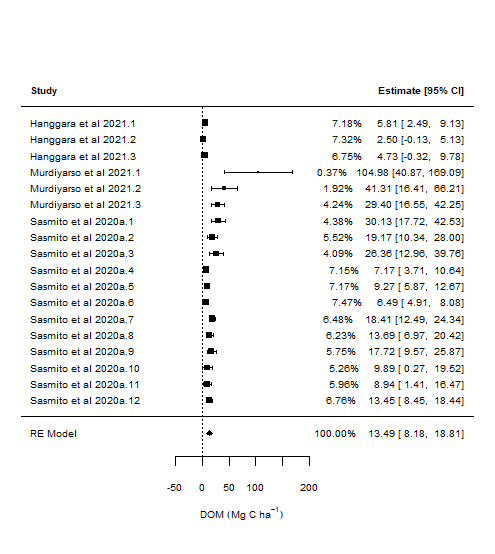


Figure S12. Random effects model results of dead organic matter carbon stocks in the regenerated mangrove


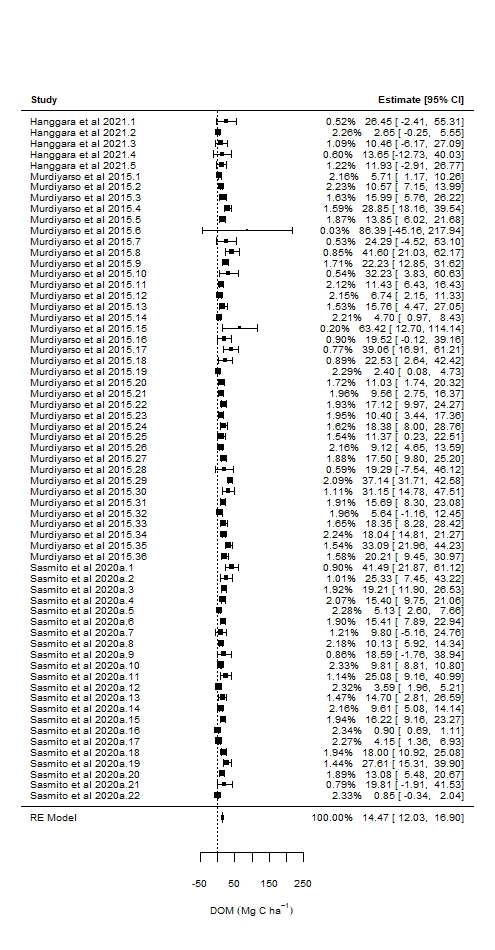


Figure S13. Random effects model results of dead organic matter carbon stocks in the undisturbed mangrove


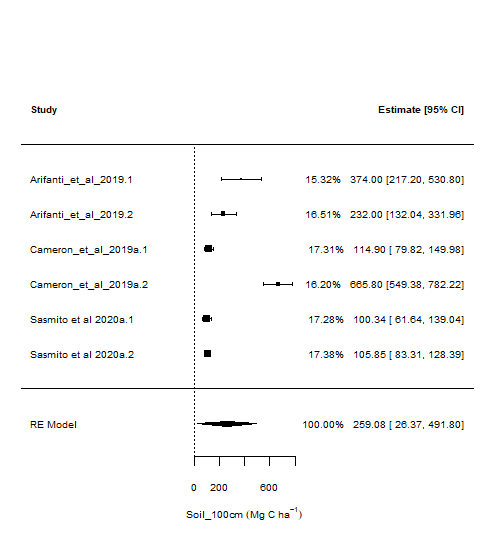


Figure S14. Random effects model results of soil 0-100cm carbon stocks in the aquaculture


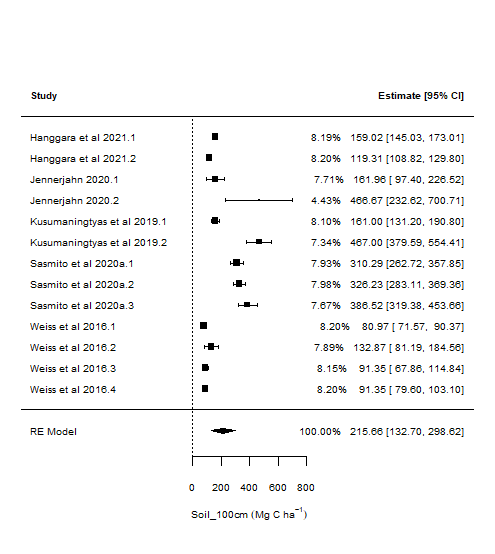


Figure S15. Random effects model results of soil 0-100cm carbon stocks in the degraded mangrove


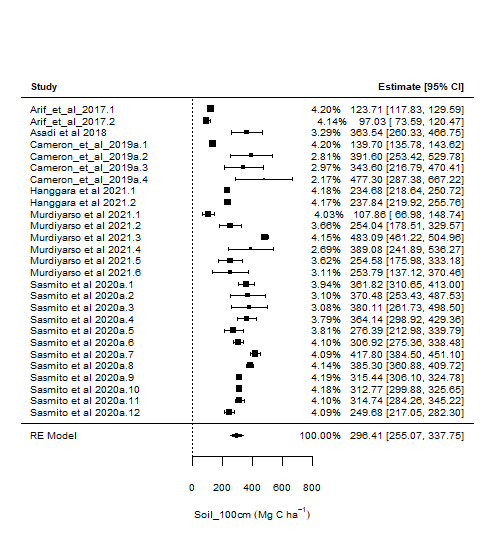


Figure S16. Random effects model results of soil 0-100cm carbon stocks in the regenerated mangrove


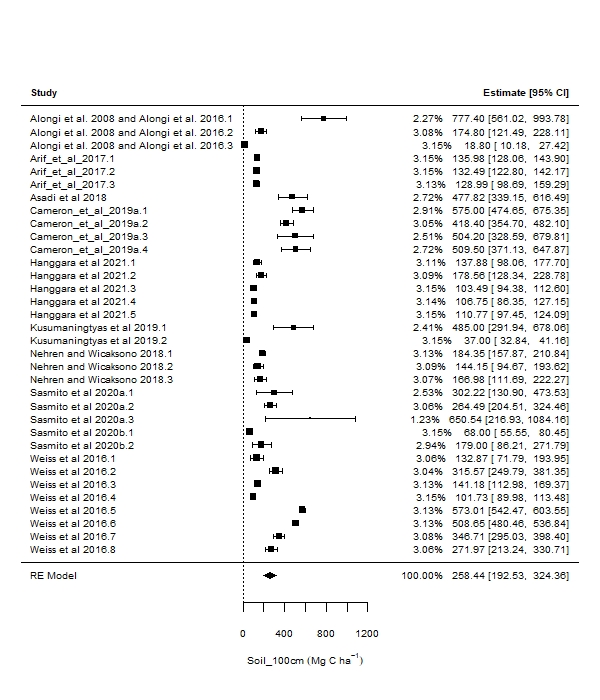


Figure S17. Random effects model results of soil 0-100cm carbon stocks in the undisturbed mangrove


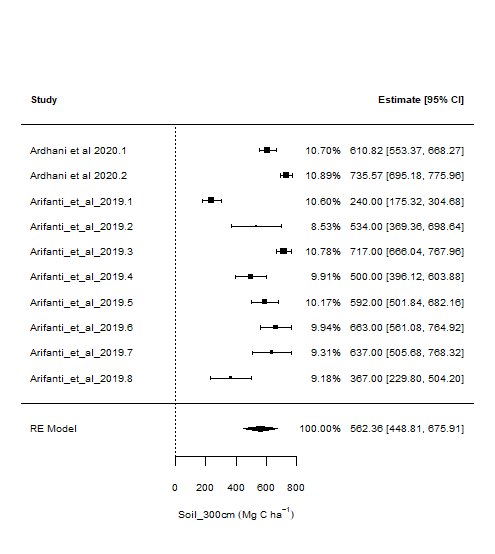


Figure S18. Random effects model results of soil 0-300cm carbon stocks in the aquaculture


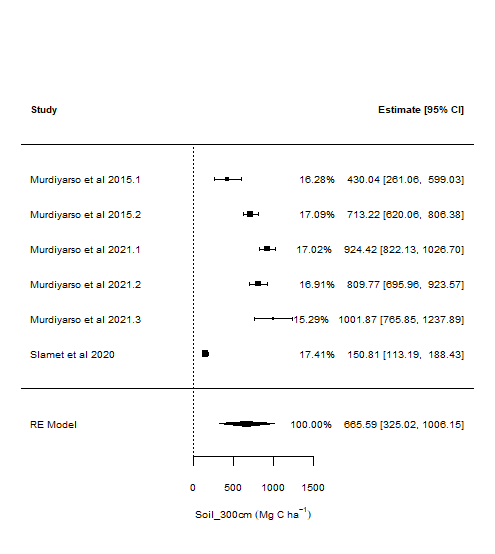


Figure S19. Random effects model results of soil 0-300cm carbon stocks in the degraded mangrove


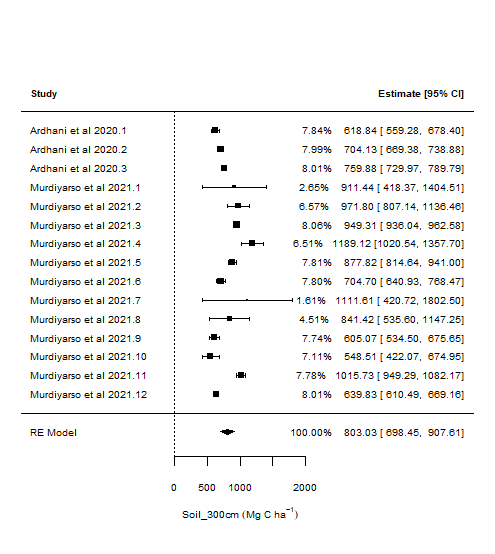


Figure S20. Random effects model results of soil 0-300cm carbon stocks in the regenerated mangrove


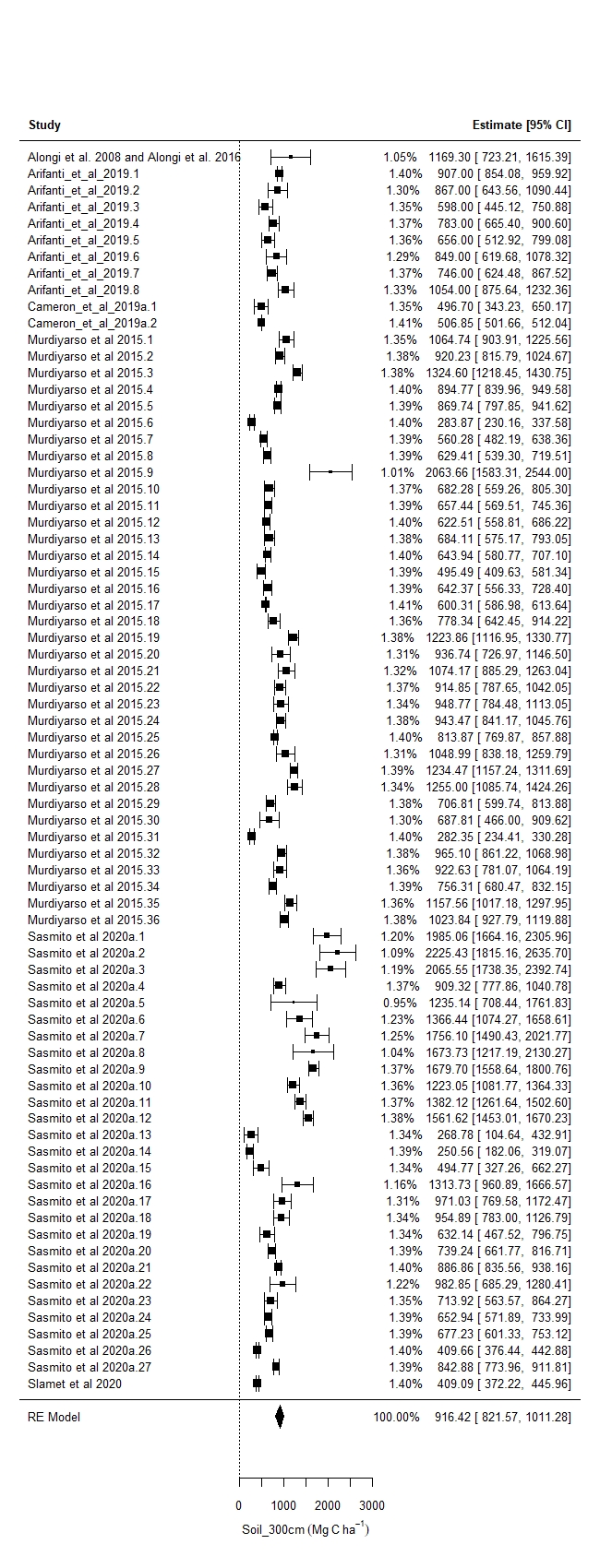


Figure S21. Random effects model results of soil 0-300cm carbon stocks in the undisturbed mangrove


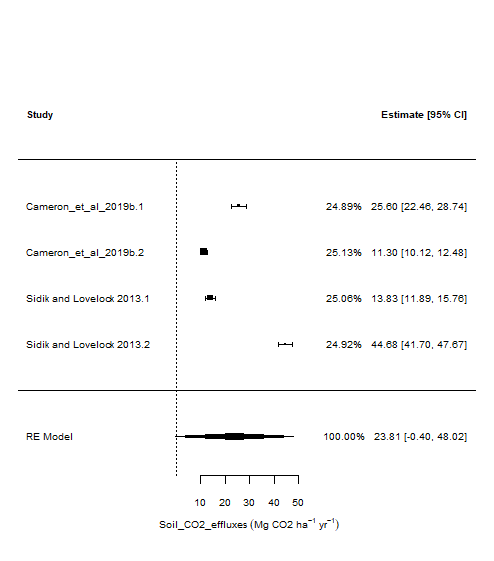


Figure S22. Random effects model results of soil CO_2_ effluxes in the aquaculture


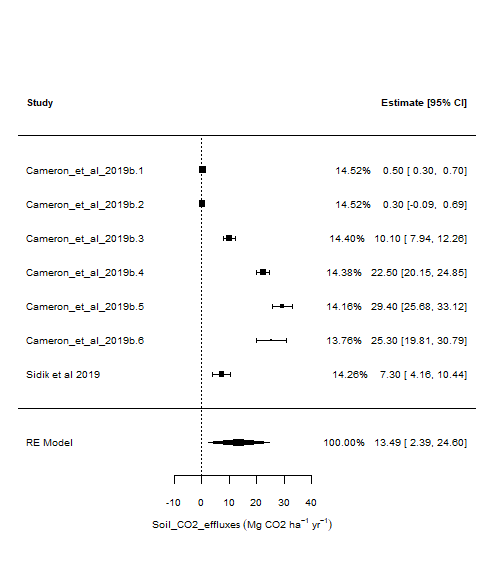


Figure S23. Random effects model results of soil CO_2_ effluxes in the regenerated mangrove


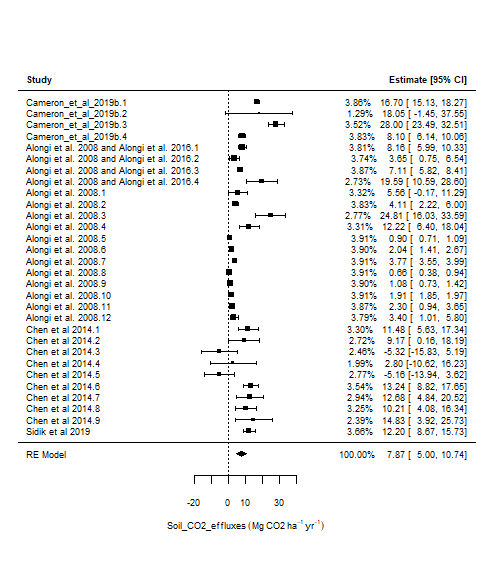


Figure S24. Random effects model results of soil CO_2_ effluxes in the undisturbed mangrove


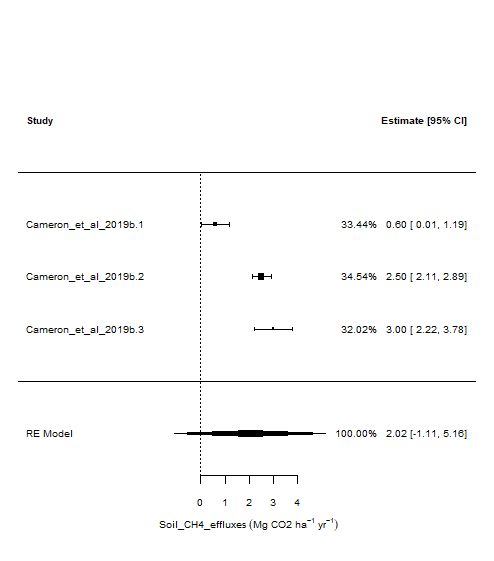


Figure S25. Random effects model results of soil CH_4_ effluxes in the aquaculture


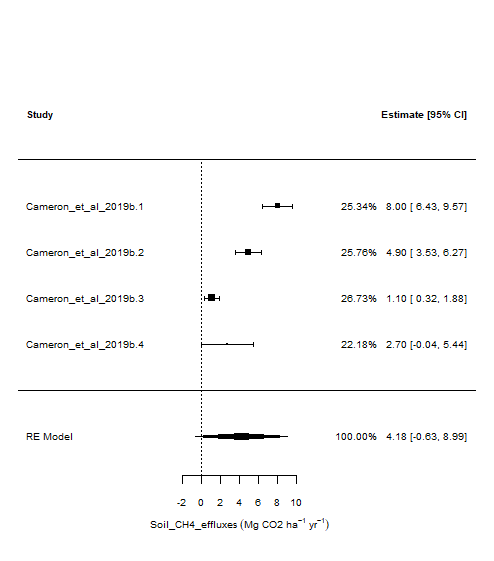


Figure S26. Random effects model results of soil CH_4_ effluxes in the regenerated mangrove


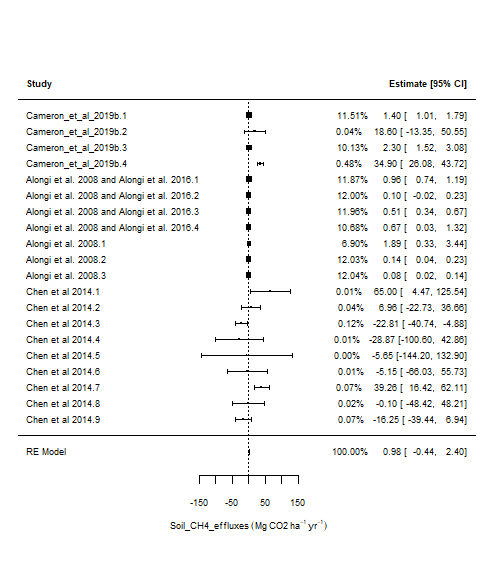


Figure S27. Random effects model results of soil CH_4_ effluxes in the undisturbed mangrove


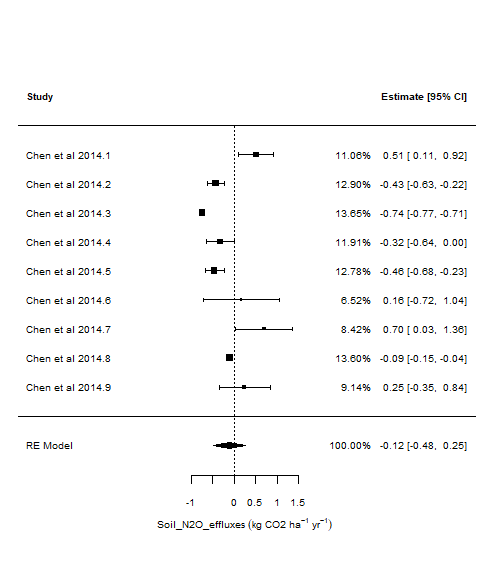


Figure S28. Random effects model results of soil N_2_O effluxes in the undisturbed mangrove


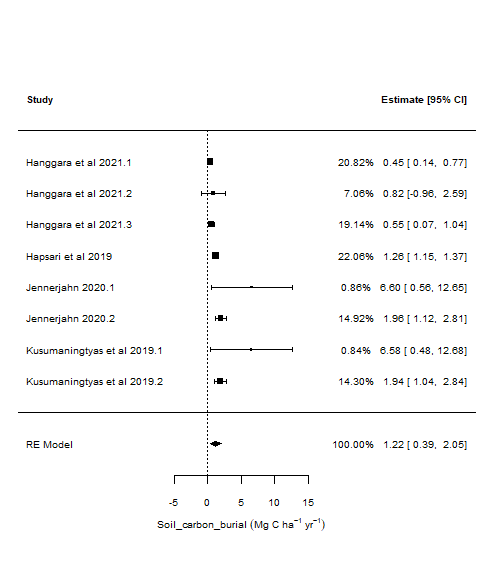


Figure S29. Random effects model results of soil carbon burial in the degraded mangrove


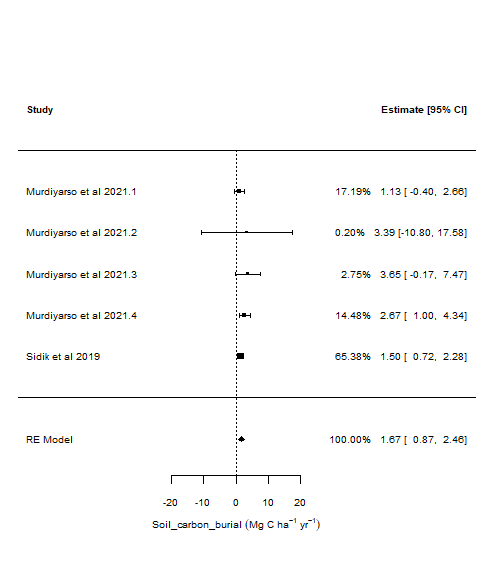


Figure S30. Random effects model results of soil carbon burial in the regenerated mangrove


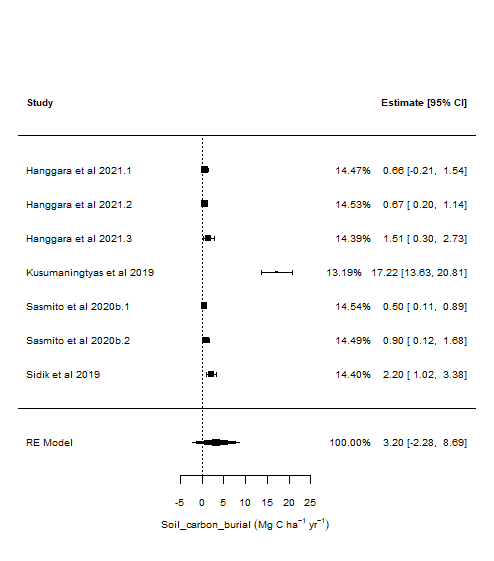


Figure S31. Random effects model results of soil carbon burial in the undisturbed mangrove

**References**

Alongi, D. M., L. A. Trott, Rachmansyah, F. Tirendi, A. D. McKinnon and M. C. Undu 2008 . "Growth and development of mangrove forests overlying smothered coral reefs, Sulawesi and Sumatra, Indonesia." Marine Ecology Progress Series 370: 97-109.

Alongi, D. M., D. Murdiyarso, J. W. Fourqurean, J. B. Kauffman, A. Hutahaean, S. Crooks, C. E. Lovelock, J. Howard, D. Herr, M. Fortes, E. Pidgeon and T. Wagey 2016 . "Indonesia's blue carbon: a globally significant and vulnerable sink for seagrass and mangrove carbon." Wetlands Ecology and Management.

Ardhani, T. S. P., MURDIYARSO, D., & KUSMANA, C. (2020). Effects of permeable barriers on total ecosystem carbon stocks of mangrove forests and abandoned ponds in Demak District, Central Java, Indonesia. Biodiversitas Journal of Biological Diversity, 21(11).

Arif, A. M., Guntur Guntur, A. B. R., Novianti, P., & Andik, I. (2017). Mangrove ecosystem C-stocks of Lamongan, Indonesia and its correlation with forest age. Research Journal of Chemistry and Environment Vol, 21, 8.

Arifanti, V. B., Kauffman, J. B., Hadriyanto, D., Murdiyarso, D., & Diana, R. (2019). Carbon dynamics and land use carbon footprints in mangrove-converted aquaculture: The case of the Mahakam Delta, Indonesia. Forest Ecology and Management, 432, 17-29. doi:10.1016/j.foreco.2018.08.047

Asadi, M. A. and G. S. Pambudi 2020 . "Diversity and biomass of mangrove forest within Baluran National park, Indonesia." AACL Bioflux 13(1

Asadi, M. A., D. Yona and M. Z. Fikri 2018 . "Comparing carbon in sediment of primary and artificially generated mangrove forests." Disaster Advances 11(11

Asadi, M. A., D. Yona and S. E. Saputro 2018 . Species Diversity, Biomass, and Carbon Stock Assessments of Mangrove Forest in Labuhan, Indonesia. IOP Conference Series: Earth and Environmental Science.

Cameron, C., Hutley, L. B., Friess, D. A., & Brown, B. (2019a). Community structure dynamics and carbon stock change of rehabilitated mangrove forests in Sulawesi, Indonesia. Ecological Applications, 29(1), e01810.

Cameron, C., Hutley, L. B., Friess, D. A., & Munksgaard, N. C. (2019b). Hydroperiod, soil moisture and bioturbation are critical drivers of greenhouse gas fluxes and vary as a function of landuse change in mangroves of Sulawesi, Indonesia. Science of the Total Environment, 654, 365-377.

Chen, G. C., Y. I. Ulumuddin, S. Pramudji, S. Y. Chen, B. Chen, Y. Ye, D. Y. Ou, Z. Y. Ma, H. Huang and J. K. Wang 2014 . "Rich soil carbon and nitrogen but low atmospheric greenhouse gas fluxes from North Sulawesi mangrove swamps in Indonesia." Science of the Total Environment 487(1

Dharmawan, I. W. E. 2018 . CO2 dynamics on three habitats of mangrove ecosystem in Bintan Island, Indonesia. Global Colloquium on Geosciences and Engineering 2017. M. M. Mukti, M. D. Yuniati and B. Setiadi. 118.

Hanggara, B. B., Murdiyarso, D., Ginting, Y. R., Widha, Y. L., Panjaitan, G. Y., & Lubis, A. A. (2021). Effects of diverse mangrove management practices on forest structure, carbon dynamics and sedimentation in North Sumatra, Indonesia. Estuarine, Coastal and Shelf Science, 107467.

Hapsari, K. A., T. C. Jennerjahn, M. C. Lukas, V. Karius and H. Behling 2019 . "Intertwined effects of climate and land use change on environmental dynamics and carbon accumulation in a mangrove-fringed coastal lagoon in Java, Indonesia." Global Change Biology.

Hidayah, Z. and L. Andriyani 2019 . Carbon Stock Analysis of Mangrove Ecosystems in Paliat Island Sumenep East Java. IOP Conference Series: Earth and Environmental Science.

Jennerjahn, T. C. (2020). Relevance and magnitude of'Blue Carbon'storage in mangrove sediments: Carbon accumulation rates vs. stocks, sources vs. sinks. Estuarine, Coastal and Shelf Science, 247, 107027.

Kangkuso, A., S. Sharma, J. Jamili, A. Septiana, I. Sahidin, U. Rianse, S. Rahim and K. Nadaoka 2018 . "Trends in allometric models and aboveground biomass of family Rhizophoraceae mangroves in the Coral Triangle ecoregion, Southeast Sulawesi, Indonesia." Journal of Sustainable Forestry 37(7

Kusumaningtyas, M. A., A. A. Hutahaean, H. W. Fischer, M. Perez-Mayo, D. Ransby and T. C. Jennerjahn 2019 . "Variability in the organic carbon stocks, sources, and accumulation rates of Indonesian mangrove ecosystems." Estuarine Coastal and Shelf Science 218: 310-323.

Malik, A., Jalil, A. R., Arifuddin, A., & Syahmuddin, A. (2020). Biomass Carbon Stocks In The Mangrove Rehabilitated Area Of Sinjai District, South Sulawesi, Indonesia. Geography, Environment, Sustainability, 13(3), 32-38.

Murdiyarso, D., J. Purbopuspito, J. B. Kauffman, M. W. Warren, S. D. Sasmito, D. C. Donato, S. Manuri, H. Krisnawati, S. Taberima and S. Kurnianto 2015 . "The potential of Indonesian mangrove forests for global climate change mitigation." Nature Climate Change 5(12

Murdiyarso, D., Sasmito, S. D., Sillanpää, M., MacKenzie, R., & Gaveau, D. (2021). Mangrove selective logging sustains biomass carbon recovery, soil carbon, and sediment. Scientific reports, 11(1), 1-10.

Nehren, U. and P. Wicaksono 2018 . "Mapping soil carbon stocks in an oceanic mangrove ecosystem in Karimunjawa Islands, Indonesia." Estuarine Coastal and Shelf Science 214: 185-193.

Rudiastuti, A. W., D. M. Yuwono and S. Hartini 2018 . Mangrove Mapping Using SPOT 6 at East Lombok Indonesia. IOP Conference Series: Earth and Environmental Science.

Sasmito, S. D., Sillanpää, M., Hayes, M. A., Bachri, S., Saragi‐Sasmito, M. F., Sidik, F., ... & Nugroho, J. D. (2020). Mangrove blue carbon stocks and dynamics are controlled by hydrogeomorphic settings and land‐use change. Global change biology, 26(5), 3028-3039.

Sasmito, S. D., Y. Kuzyakov, A. A. Lubis, D. Murdiyarso, L. B. Hutley, S. Bachri, D. A. Friess, C. Martius and N. Borchard 2020 . "Organic carbon burial and sources in soils of coastal mudflat and mangrove ecosystems." Catena 187.

Sidik, F. and C. E. Lovelock 2013 . "CO2 Efflux from Shrimp Ponds in Indonesia." PLoS ONE 8(6

Sidik, F., M. F. Adame and C. E. Lovelock 2019 . "Carbon sequestration and fluxes of restored mangroves in abandoned aquaculture ponds." Journal of the Indian Ocean Region 15(2

Slamet, N. S., Dargusch, P., Aziz, A. A., & Wadley, D. (2020). Mangrove vulnerability and potential carbon stock loss from land reclamation in Jakarta Bay, Indonesia. Ocean & Coastal Management, 195, 105283.

Weiss, C., J. Weiss, J. Boy, I. Iskandar, R. Mikutta and G. Guggenberger 2016 . "Soil organic carbon stocks in estuarine and marine mangrove ecosystems are driven by nutrient colimitation of P and N." Ecology and Evolution 6(14
